# Hyperferritinaemia in Dengue Virus Infected Patients Is Associated with Immune Activation and Coagulation Disturbances

**DOI:** 10.1371/journal.pntd.0003214

**Published:** 2014-10-09

**Authors:** Cornelia A. M. van de Weg, Ralph M. H. G. Huits, Cláudio S. Pannuti, Rosalba M. Brouns, Riemsdijk W. A. van den Berg, Henk-Jan van den Ham, Byron E. E. Martina, Albert D. M. E. Osterhaus, Mihai G. Netea, Joost C. M. Meijers, Eric C. M. van Gorp, Esper G. Kallas

**Affiliations:** 1 Department of Viroscience, Erasmus Medical Center, Rotterdam, The Netherlands; 2 Emergency Department and Department of Internal Medicine, Dr. Horacio E. Oduber Hospitaal, Oranjestad, Aruba; 3 Instituto de Medicina Tropical de São Paulo e Departamento de Moléstias Infecciosas e Parasitárias (LIM-52), Faculdade de Medicina, Universidade de São Paulo, São Paulo, Brazil; 4 Landslaboratorium, Oranjestad, Aruba; 5 Department of Experimental Internal Medicine, Radboud University, Nijmegen, The Netherlands; 6 Department of Experimental Vascular Medicine, Academic Medical Center, Amsterdam, The Netherlands; 7 Disciplina de Imunologia Clínica e Alergia (LIM-60), Faculdade de Medicina, Universidade de São Paulo, São Paulo, Brazil; University of Pittsburgh, United States of America

## Abstract

**Background:**

During a dengue outbreak on the Caribbean island Aruba, highly elevated levels of ferritin were detected in dengue virus infected patients. Ferritin is an acute-phase reactant and hyperferritinaemia is a hallmark of diseases caused by extensive immune activation, such as haemophagocytic lymphohistiocytosis. The aim of this study was to investigate whether hyperferritinaemia in dengue patients was associated with clinical markers of extensive immune activation and coagulation disturbances.

**Methodology/Principal Findings:**

Levels of ferritin, standard laboratory markers, sIL-2R, IL-18 and coagulation and fibrinolytic markers were determined in samples from patients with uncomplicated dengue in Aruba. Levels of ferritin were significantly increased in dengue patients compared to patients with other febrile illnesses. Moreover, levels of ferritin associated significantly with the occurrence of viraemia. Hyperferritinaemia was also significantly associated with thrombocytopenia, elevated liver enzymes and coagulation disturbances. The results were validated in a cohort of dengue virus infected patients in Brazil. In this cohort levels of ferritin and cytokine profiles were determined. Increased levels of ferritin in dengue virus infected patients in Brazil were associated with disease severity and a pro-inflammatory cytokine profile.

**Conclusions/Significance:**

Altogether, we provide evidence that ferritin can be used as a clinical marker to discriminate between dengue and other febrile illnesses. The occurrence of hyperferritinaemia in dengue virus infected patients is indicative for highly active disease resulting in immune activation and coagulation disturbances. Therefore, we recommend that patients with hyperferritinaemia are monitored carefully.

## Author Summary

Ferritin is an acute-phase reactant and produced by reticulo-endothelial cells in response to inflammation and infection. In general, ferritin levels are increased in inflammatory conditions, but in this study we found that ferritin levels were much higher in dengue virus infected patients than in patients with other febrile illnesses. This indicates that ferritin could be used as a marker to discriminate between dengue and other febrile diseases. Moreover, the presence of hyperferritinaemia (ferritin levels≥500 µg/L) was associated with markers of immune activation and coagulation disturbances and clinical disease severity, suggesting that it could serve as a marker of activity of disease. Clinical markers to determine the presence and severity of dengue virus infection are important for diagnostic and treatment purposes. Our results indicate that increased ferritin levels could be used to increase the likelihood on a positive dengue diagnosis. Moreover, patients with hyperferritinaemia should be monitored carefully, because they are at risk to develop severe disease due to extensive immune activation.

## Introduction

Outbreaks of dengue virus (DENV) infection have become more frequent in the American and Caribbean region, even threatening to spread in the United States [1]. DENV is a flavivirus, which is transmitted by the bite of an Aedes mosquito. Brazil is the country with most reported dengue cases in the Americas. A large DENV-2 outbreak in 2010 caused more than 34.000 cases and 64 deaths in the State of São Paulo, Brazil [2]. On the Caribbean island Aruba, there was an epidemic from September 2011 till April 2012, in which DENV-1 and DENV-4 were both co-circulating.

The symptoms of DENV infection are mild and self-limiting in the majority of cases, consisting of fever, headache, retro-orbital pain, myalgia, arthralgia, thrombocytopenia, minor mucosal bleeding and skin manifestations. Some patients develop severe symptoms, such as shock, severe bleeding or organ impairment. These symptoms usually develop three to five days after the onset of disease around the time of defervescence. It has been hypothesized that severe dengue is caused by a cytokine storm inducing systemic inflammatory effects (Reviewed in [3]). The pathophysiological mechanisms that cause this cytokine storm are not fully unravelled and represent an important focus for dengue research.

In addition to the current laboratory markers for dengue, ferritin levels were described to be associated with clinical disease severity in children [4]. In many cases ferritin levels higher than 500 µg/L were detected, defined as hyperferritinaemia [5]. Ferritin is an acute-phase reactant and highly expressed by cells of the reticulo-endothelial system in response to infection and inflammation. Ferritin binds iron, limiting its availability in the circulation. Because many pathogenic microorganisms need iron for their proliferation, this mechanism is favourable for the host. Moreover, iron deficiency enhances the immunological performance of lymphocytes, neutrophils and macrophages (reviewed in [6]).

Hyperferritinaemia is a hallmark of diseases, characterized by extensive immune activation, including haemophagocytic lymphohistiocytosis (HLH) and macrophage activation syndrome (MAS). HLH can be congenital or triggered by an external stimulus, such as malignancy or viral infection, including dengue [7]. NK cells and CD8+ T lymphocytes are impaired in their cytotoxic function in patients with HLH, which results in reduced clearance of infected and antigen-presenting cells from the circulation. This may lead to an exaggerated immune response with proliferation of dendritic cells, tissue macrophages and T-cells, contributing to a cytokine storm (reviewed in [8]). The symptoms of HLH consist of ongoing fever, hepatosplenomegaly, cytopenia (affecting more than 2 cell lineages), hypofibrinogenaemia, hypertriglyceridaemia, hyperferritinaemia, increased levels of sIL-2R and coagulopathy (reviewed in [5]).

Although clinical symptoms of DENV infection are usually rather mild compared to HLH, some patients develop severe symptoms. These are most probably caused by extensive immune activation and show similarities with the clinical hallmarks of HLH and MAS, suggesting similar pathophysiology.

The aim of this study was to investigate the association between hyperferritinaemia, immune activation and coagulation disturbances in DENV infected patients. We showed that the presence of hyperferritinaemia could discriminate between dengue and other febrile diseases. Moreover, we found an association between increased ferritin levels and severe clinical disease, thrombocytopenia, liver enzyme and coagulation disturbances and a pro-inflammatory cytokine profile.

## Materials and Methods

### Cohort Aruba and Brazil

#### Ethics statement

The study in Aruba was approved by the Institutional Review Board of the Dr. Horacio Oduber Hospitaal in Aruba. The study in Brazil was approved by the Institutional Review Board from Hospital das Clínicas, University of São Paulo (CAPPesq - Research Projects Ethics Committee) under protocol 0652/09. Patients in both studies were included after written informed consent was obtained. In case of participants younger than 18 years informed consent was obtained from their parent or legal guardian. Clinical data and blood samples were anonymized with a study number.

In Aruba, all patients of 16 years and older with a clinical suspicion of dengue, presenting at the emergency room of the Dr. Horacio Oduber Hospitaal and one clinical practice between September 2011 and April 2012, were included. Pregnant women and patients with no available data about clinical symptoms were excluded. After obtaining informed consent, the treating physician filled out a standard case report form (CRF). Patients were admitted to the hospital when at least one of the following symptoms was present: systolic blood pressure lower than 90 mm Hg, diastolic blood pressure lower than 60 mmHg, pulse rate higher than 100/min, signs of dehydration, platelet count lower than 50.000 cells/mm^3^, increase in haematocrit above 50%, or signs of mucosal bleeding. Patients were classified according to the 2009 WHO dengue case classification [9], [10]. Briefly, patients with fever and general symptoms were classified as non-severe dengue without warning signs (WS−). Patients with one of the following warning signs were classified as non-severe dengue with warning signs (WS+): abdominal pain, vomiting, minor mucosal bleeding, pleural effusion, ascites and hepatomegaly. Patients with shock, respiratory distress, severe bleeding and/or organ impairment were classified as severe dengue.

Serum and plasma samples were drawn at day 2–3, 4–5 and 6–8 and 28 days after the onset of fever. Samples not immediately used, were stored at −80°C and shipped to The Netherlands on dry ice. Repetitive freeze-thaw cycles were avoided. The samples from day 28 were used as an autologous control group.

The clinical diagnosis of dengue was confirmed either by a positive NS1 antigen test (‘Platelia’, Bio-Rad, France) at day 2–3 and/or a positive IgM enzyme-linked immunosorbent assay (ELISA) (‘IgM Capture DxSelect’, Focus Diagnostics, USA) at day 4–5 and/or an increase in IgG titre (‘IgG Capture DxSelect’, Focus Diagnostics, USA) between the acute and convalescent sample. Patients with a negative NS1, IgM and IgG in the acute phase sample and a negative IgG in the convalescent sample were diagnosed as Other Febrile Illness (OFI). If the IgG was positive in the acute phase sample, but the convalescent sample was missing, patients were considered inconclusive and excluded from this analysis. Patients with a positive IgG in the sample from day 4–5 were considered as secondary DENV infection, while patients with a negative IgG in this sample were considered as a primary DENV infection [11].

The cohort in Brazil was described previously [12]. Briefly, during the 2010 DENV-2 outbreak, patients with clinical suspected dengue fever presenting at the Ana Costa Hospital, Santos, State of São Paulo were included. Patients were diagnosed with DENV infection by detection of DENV NS1 antigen and/or IgM-specific antibodies using a commercially available rapid test (Dengue Duo Test Bioeasy, Standard Diagnostic Inc. 575-34, Korea) or by detection of DENV RNA by real time PCR (RT-PCR). Serum samples were drawn and stored at −80°C. Patients were classified according to the 2009 WHO classification and the occurrence of haemorrhagic manifestations and the occurrence of plasma leakage and shock [9], [10]. Age-matched healthy volunteers with a similar socio-economic background were used as controls.

### Serotype- and copy number quantitative reverse transcriptase PCR (RT-PCR) on serum samples from Aruba

In order to determine the infecting serotype a semi-quantitative RT-PCR (Taqman) was performed. Primers and probes directed against the capsid were derived from Sadon et al. [13]. Briefly, 4× TaqMan Fast Virus 1-step Master Mix (Invitrogen) was used with 20 pmol of primers and 10 pmol of probes. The cycling program consisted of 5 minutes at 50°C, then 20 seconds at 95°C followed by 40 cycles of 3 seconds at 95°C and 30 seconds at 60°C.

Another quantitative RT-PCR was performed to determine the viral copy number. The primers and probes directed against the 3′UTR were derived from Drosten et al. [14]. Briefly, 4× TaqMan Fast Virus 1-step Master Mix was used with 15 pmol of primers and 10 pmol of probes and an additional 25 mM of MgCl_2_ was added [15]. The cycling program was similar to the serotype quantitative RT-PCR.

### Ferritin and cytokines Aruba

Plasma ferritin concentrations were determined at the Landslaboratorium in Aruba within a few hours after blood sampling. The assay was performed using the ‘Access’ (Beckman Coultier, USA) under standardized conditions.

Serum sIL-2Rα and IL-18 levels were determined at the department of experimental internal medicine from the Radboud University. sIL-2Rα was measured using a commercially available luminex kit (‘Milliplex’, Merck Millipore, Germany). Samples were diluted 1∶5 and the assay was performed according to the manufacturer's instructions and run on a Luminex 200 dual laser detection system. The sensitivity limit was 15 pg/ml. Levels of IL-18 were measured using a commercially available ELISA kit (MBL, Japan) according to the manufacturer's instructions.

### Markers of coagulation Aruba

All markers of coagulation were determined in citrate samples.

Activated partial thromboplastin time (APTT) and prothrombin time (PT) were determined at the Landslaboratorium in Aruba within a few hours after blood withdrawal. PT (Dade Innovin) and APTT (Dade Actin FSL) were determined on a Sysmex CA-1500 System (Siemens Healthcare Diagnostics, USA).

All other coagulation parameters were determined at the department of Experimental Vascular Medicine from the Academic Medical Centre. Von Willebrand Factor (vWF) was measured using a home-made ELISA with antibodies from DAKO (Glostrup, Denmark). In vitro thrombin generation was assayed by measuring peak thrombin levels with the Calibrated Automated Thrombography (CAT) as described previously [16]. In vivo thrombin generation was determined by detecting thrombin-antithrombin complexes (TAT) using a commercially available ELISA (Enzygnost). Levels of the fibrinolytic markers plasminogen activator inhibitor type 1 (PAI-1) and plasmin-α2-antiplasmin (PAP) complexes were measured with commercially available ELISAs according to the manufacturer's instructions (PAI-1, Hyphen BioMed; PAP complexes, DRG Diagnostics). D-dimer levels were determined with a particle-enhanced immunoturbidimetric assay (Innovance D-dimer, Siemens Healthcare Diagnostics).

### Ferritin and cytokine assays Brazil

Serum ferritin levels were determined with a commercially available ELISA (Biolisa ferritina, Bioclin, Brazil) performed according to the manufacturer's instructions.

The measurement of cytokines and the cluster analysis have been described in a previous publication [12]. Briefly, levels of thirty cytokines were measured using a multiplex immunoassay kit with spectrally encoded antibody-conjugated beads (Human Cytokine 30-plex panel, Invitrogen, USA). Twenty-three cytokines were used in a cluster analysis procedure, which was adapted from van den Ham et al. [17]. Briefly, cytokine values were log-transformed and subjected to hierarchical correlation clustering (i.e., with distance measure 1 – pearson's pairwise correlation value) using Ward's method.

### Statistics

IBM SPSS Statistics v.20 was used to calculate statistical significance. The Mann Whitney U test was used to compare the difference between two groups. The Spearman's correlation coefficient was applied to calculate correlations. The Chi-Squared test was used to calculate differences in proportions between groups and the Fisher's exact to determine whether one distribution was unequally distributed over the groups. Using bonferroni correction the p-value was adjusted for multiple testing.

## Results

Seventy-three patients were included in Aruba between September 2011 and April 2012. The clinical diagnosis of forty-four patients could be confirmed by serology and/or RT-PCR (Table 1). Seventeen patients tested negative for DENV infection and were included in the OFI group. Twelve patients were excluded with an inconclusive diagnosis. In that particular season, both DENV-1 and DENV-4 were circulating. One patient tested positive for DENV-2, but this patient was probably infected in Suriname. Moreover, 11 patients suffered from a primary and 30 patients from a secondary infection. In three patients the infection status could not be determined.

**Table 1:** General characteristics cohort Aruba.

	WS−	WS+	Statistics	No hyper- ferritinaemia	Hyper-ferritinaemia	Statistics	OFI
Number of patients	17	27		24	19		17
Age*	49 (30–57)	42 (31–63)		40 (30–55)	49 (32–64)		35 (29–52)
Sex	8 male (47%)	10 male (37%)	P = 0.6 (F)	7 male (29%)	11 male (58%)	P = 0.07 (F)	6 male (35%)
Infection status	2 (12%) indeterminate, 6 (35%) primary, 9 (53%) secondary	1 (4%) indeterminate, 5 (19%) primary, 21 (78%) secondary	P = 0,2 (Chi)	2 (8%) indeterminate, 9 (38%) primary, 13 (54%) secondary	1 (5%) indeterminate, 2 (11%) primary, 16 (84%) secondary	P = 0,1 (Chi)	NA
Viraemic	10 (59%)	18 (67%)	P = 1.0 (F)	12 (50%)	16 (84%)	P = 0.05 (F)	NA
Serotype	5 DENV-1, 3 DENV-4	3 DENV-1, 1 DENV-2, 8 DENV-4		5 DENV-1, 2 DENV-4	3 DENV-1, 1 DENV-2, 9 DENV-4		NA
Hospitalization rate	4 (24%)	11 (41%)	P = 0.3 (F)	6 (25%)	8 (42%)	P = 0.3 (F)	2 (12%)

In one patient ferritin levels were not determined. Abbreviations: WS− = non-severe dengue without warning signs. WS+ = non-severe dengue with warning signs, OFI = other febrile illness, F = Fisher's exact test, Chi = Chi-squared test, DENV = dengue virus.

* = Values are in median (interquartile range).

The epidemic was rather mild and only one case of severe dengue was recorded according to the 2009 WHO dengue case classification. This patient presented with melaena and was included in the WS+ group. From the other dengue positive patients, seventeen were classified as WS− and twenty-six as WS+ dengue. The most common warning sign was abdominal pain (20/44, 45%) followed by vomiting (10/44, 23%) and in a few patients epistaxis (2/44, 5%) and hepatomegaly (1/44, 2%) was reported. Pleural effusion and ascites were not reported, probably because ultrasound and/or X-ray examination were performed on a limited basis. Fifteen dengue patients were admitted to the hospital.

A total of 191 sequential samples from the forty-four patients from Aruba were included in this analysis collected at day 2–8 after the onset of fever (sample size in Table S1). Follow-up samples collected at day 28 from dengue and OFI patients served as an autologous control group.

### Ferritin expression is associated with dengue virus infection and viraemia

**Figure 1:**
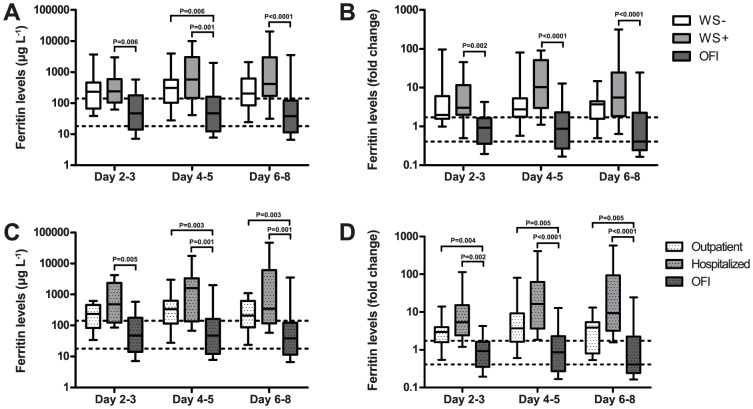
Ferritin levels and fold change are increased in dengue patients from Aruba. Absolute levels of ferritin were significantly increased in WS+ patients compared to OFI at each time point and in WS− patients compared to OFI at day 4–5 (A). Absolute levels were also elevated in hospitalized and outpatients compared to OFI (C) at almost each time point. The ferritin fold change was significantly elevated in WS+ patients compared to OFI (B) and in hospitalized and outpatients compared to OFI (D) at each time point. Area between two dotted horizontal lines: interquartile range from the autologous control group. Boxplots indicate the interquartile range, the horizontal line inside the box indicates the median. The whiskers reach from the 10^th^ till the 90^th^ percentile. P-value≤0.006 is considered significant.

Ferritin levels were determined in patients with dengue and OFI to identify any association with disease severity. Using the 2009 WHO dengue case classification, ferritin levels were significantly increased in WS+ patients compared to OFI at each time point and in WS− patients compared to OFI at day 4–5 (Figure 1A). At day 4–5 and 6–8 the highest ferritin levels were observed and a tendency was shown towards higher ferritin levels in WS+ patients compared to WS−, although these differences were not statistically significant.

In clinical practice and according to the official HLH-criteria, ferritin levels ≥500 µg/L are considered hyperferritinaemia [5]. A larger proportion of males showed hyperferritinaemia compared to females in this cohort, which approached statistical significance (Table 1). It is known that baseline ferritin levels are higher in the male than in the female population [18]. We calculated a fold change by dividing the absolute values of ferritin by the median ferritin levels for males and females from the autologous control group (Females:/37 µg/L and males:/154 µg/L), which were similar to levels previously described [18]. The ferritin fold change was significantly increased in WS+ dengue patients compared to OFI at each time point (Figure 1B).

Another marker of disease severity is the hospitalization rate. Absolute levels and the ferritin fold change were significantly increased in hospitalized and outpatients compared to OFI at almost all time points (Figure 1C and 1D). The absolute ferritin levels as well as the fold change showed a tendency of increased values in hospitalized patients compared to outpatients.

Because the difference in ferritin levels between patients with dengue and OFI was significant, we calculated an odds ratio for the occurrence of hyperferritinaemia and a confirmed diagnosis of DENV infection. In dengue patients, 19 out of 43 had hyperferritinaemia compared to two out of 17 patients with OFI. This resulted in a sensitivity of 44%, a specificity of 88% and an odds ratio of 6. The high values of the specificity and odds ratio suggest that the occurrence of hyperferritinaemia may serve as a discriminatory marker between dengue and OFI.

The presence or absence of viraemia in the early phase was linked to ferritin levels during the course of disease. Patients were considered viraemic if they had detectable virus titres at day 2–3 and day 4–5. Patients with undetectable levels at these days were considered non-viraemic.

**Figure 2:**
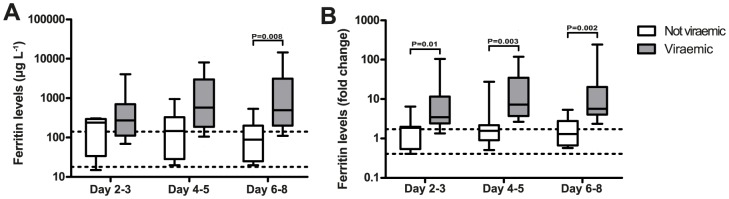
Ferritin levels and fold change are associated with viraemia. The absolute ferritin levels were significantly increased in viraemic patients compared to non-viraemic patients at day 6–8 (A) and the ferritin fold change was significantly elevated in viraemic patient at each time point (B) in the cohort from Aruba. Area between two dotted horizontal lines: interquartile range from the autologous control group. Boxplots indicate the interquartile range. The horizontal line inside the box indicates the median. The whiskers reach from the 10^th^ till the 90^th^ percentile. P-value≤0.02 is considered significant.

The absolute ferritin levels were significantly elevated in viraemic patients compared to non-viraemic patients at day 6–8 (Figure 2A). The ferritin fold change was significantly elevated in viraemic patients at all time points (Figure 2B). There were no strong correlations between the viral load and the levels of ferritin at the same day of disease. However, absolute levels of ferritin at day 6–8 correlated significantly with the viral copy number at day 2–3 (ρ = 0.5; P = 0.008) and day 4–5 (ρ = 0.5; P = 0.002) (Figure S1). The ferritin fold change at day 6–8 also showed a significant correlation with the viral load at day 2–3 (ρ = 0.5; P = 0.003) and day 4–5 (ρ = 0.6; P<0.0001). This suggests that viral replication in the early phase of disease may cause an increase in ferritin levels in the convalescent phase.

### Hyperferritinaemia in dengue is associated with thrombocytopenia and elevated liver enzymes

Hyperferritinaemia is a prominent symptom of patients with HLH. To investigate whether the clinical picture of DENV infection shows more similarities, the official diagnostic criteria for HLH [5] were linked to hyperferritinaemia in dengue patients. In each patient the occurrence of hyperferritinaemia was evaluated at each time point.

**Figure 3:**
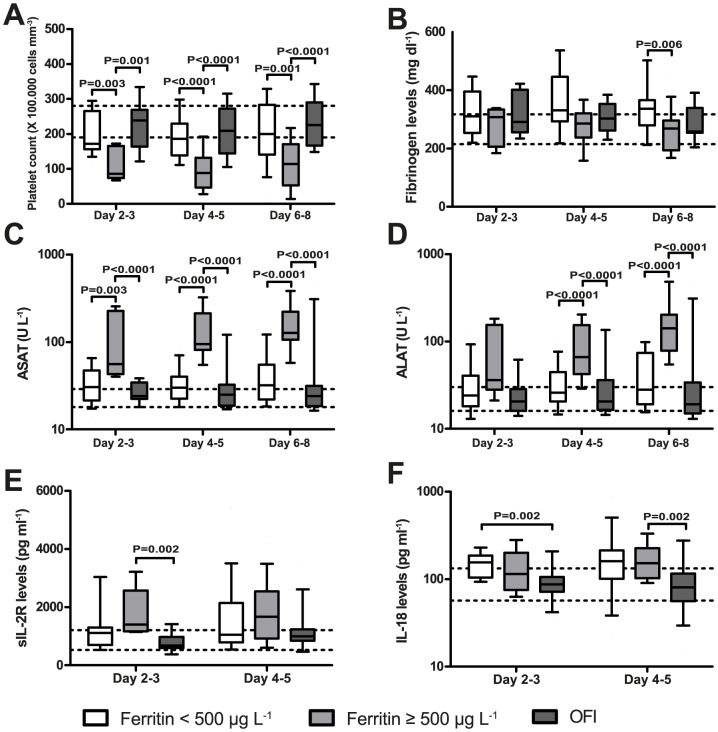
Hyperferritinaemia is associated with certain markers of HLH and MAS. The platelet count (A) was significantly decreased and the liver enzyme ASAT (C) significantly increased in patients with hyperferritinaemia compared to patients with no hyperferritinaemia and OFI at each time point in the cohort from Aruba. Levels of fibrinogen were significantly decreased in patients with hyperferritinaemia compared to patients without hyperferritinaemia at day 6–8 (B). Levels of ALAT were significantly increased in patients with hyperferritinaemia at day 4–5 and 6–8 (D). Levels of sIL-2R were significantly increased in patients with hyperferritinaemia compared to OFI at day 2–3 (E). Levels of IL-18 were significantly increased in patients with no hyperferritinaemia compared to OFI at day 2–3 and in patients with hyperferritinaemia compared to OFI at day 4–5 (F). Missing values: Platelet count, ASAT, ALAT: no missing values. sIL-2R and IL-18: Day 2–3: No HF (N = 3), HF (N = 2). Day 4–5: No HF (N = 5), HF (N = 3). Area between two dotted horizontal lines: interquartile range from the autologous control group. Boxplots indicate the interquartile range, the horizontal line inside the box indicates the median. The whiskers reach from the 10^th^ till the 90^th^ percentile. P-value≤0.006 is considered significant.

Severe cytopenia in at least two cell lineages is a prominent feature of HLH due to the increased phagocytic activity of macrophages. In our cohort the platelet count was significantly decreased in patients with hyperferritinaemia compared to patients with no hyperferritinaemia and OFI at each time point (Figure 3A, Table S2). No significant differences in the leukocyte count were detected (Data not shown).

Another criterium is the presence of hypertriglyceridaemia and/or hypofibrinogenaemia. Levels of fibrinogen were significantly decreased in patients with hyperferritinaemia compared to patients without hyperferritinaemia at day 6–8, but levels were still in the range of the autologous control group (Figure 3B). The triglyceride levels were in the normal range of the autologous control group in both dengue as well as OFI patients (data not shown).

MAS is characterized by hepatosplenomegaly and liver dysfunction. The liver also plays an important role in the pathogenesis of DENV infection. Levels of the liver enzyme ASAT were significantly increased in patients with hyperferritinaemia compared to patients with no hyperferritinaemia and OFI at each time point (Figure 3C). ALAT levels were also significantly increased in patients with hyperferritinaemia at day 4–5 and 6–8 (Figure 3D).

sIL-2R is a marker of T-cell activation and IL-18 of macrophage activation. sIL-2R was significantly increased in patients with hyperferritinaemia compared to OFI at day 2–3 (Figure 3E). Levels of IL-18 were significantly elevated in patients with no hyperferritinaemia compared to OFI at day 2–3 and in patients with hyperferritinaemia compared to OFI at day 4–5 (Figure 3E). Altogether, we can conclude that hyperferritinaemia in uncomplicated dengue patients is strongly associated with thrombocytopenia and elevated liver enzymes, but these patients had no hypertriglyceridaemia, hypofibrinogenaemia or cytopenia in another lineage than the platelets.

### Hyperferritinaemia is associated with activation of coagulation and fibrinolysis

**Figure 4:**
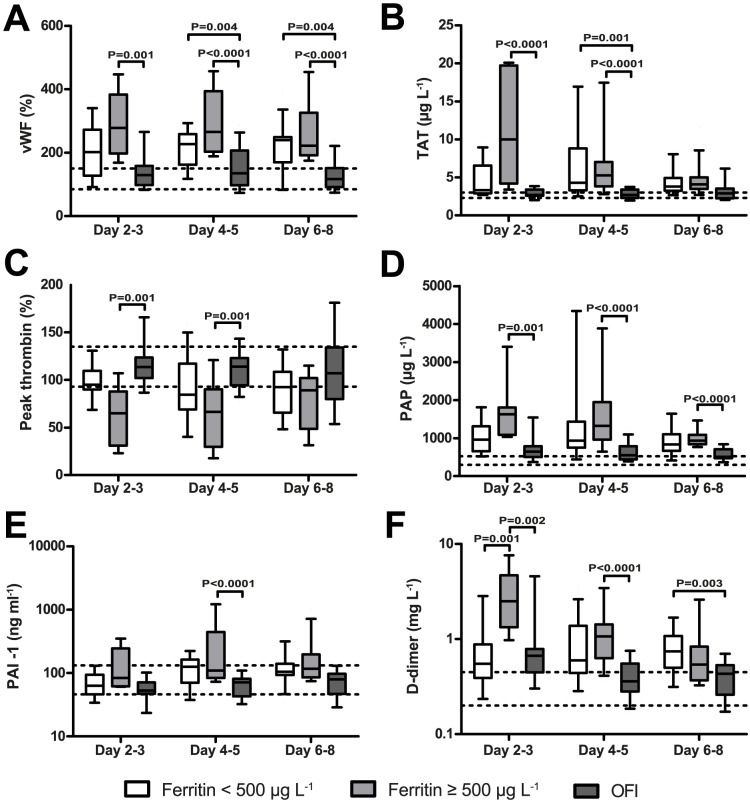
Hyperferritinaemia is associated with markers of coagulation and fibrinolysis. Levels of vWF were significantly increased in patients with hyperferritinaemia compared to OFI at each time point and significantly increased in patients without hyperferritinaemia compared to OFI at day 4–5 and 6–8 (A) in the cohort from Aruba. Levels of TAT (B) were significantly increased and levels of peak thrombin (C) significantly decreased in patients with hyperferritinaemia at day 2–3 and 4–5. Levels of PAP were significantly increased in patients with hyperferritinaemia compared to OFI (D). Levels of PAI-1 were significantly increased in patients in patients with hyperferritinaemia compared to OFI at day 4–5 (E). Levels of D-dimer were significantly increased in patients with hyperferritinaemia compared to OFI at day 2–3 and 4–5 and in patients with hyperferritinaemia compared to patients without hyperferritinaemia at day 2–3 (F). Missing values vWF, PAI-1: Day 2–3: OFI (N = 1). Day 4–5: HF (N = 1); TAT, PAP, D-dimer: No missing values; Peak thrombin: Day 2–3: No (N = 1), OFI (N = 1). Day 4–5: No (N = 1), HF (N = 4), OFI (N = 2). Day 6–8: No (N = 2), HF (N = 1), OFI (N = 2). Area between two dotted horizontal lines: interquartile range from the autologous control group. Boxplots indicate the interquartile range, the horizontal line inside the box indicates the median. The whiskers reach from the 10^th^ till the 90^th^ percentile. P-value≤0.006 is considered significant.

Hyperferritinaemia was investigated in association with parameters, indicating the activation of coagulation and fibrinolysis. The APTT and PT showed no significant differences between any of the groups (data not shown). vWF is released upon endothelial cell activation and plays an important role in the formation of the thrombus. Significantly increased levels were found in patients with hyperferritinaemia compared to OFI at day 2–3 and in both dengue groups compared to OFI at day 4–5 and 6–8 (Figure 4A). Activation of the coagulation cascade starts with thrombin generation after which it is bound by antithrombin. Thrombin-antithrombin (TAT) complexes are a marker for activation of the coagulation cascade in vivo. Levels were significantly elevated in dengue patients with hyperferritinaemia compared to OFI at day 2–3 and 4–5 and also in patients without hyperferritinaemia compared to OFI at day 4–5 (Figure 4B). The ability of plasma to generate thrombin in vitro can be investigated by the calibrated automated thrombrogram measuring peak thrombin levels. Interestingly, while the levels of TAT were significantly increased, the peak thrombin levels were significantly decreased in patients with hyperferritinaemia compared to OFI at day 2–3 and 4–5 (Figure 4C). Thrombin generation will lead to fibrin formation and eventually fibrinolysis, resulting in the formation of plasminogen-α2-antiplasmin (PAP) complexes. PAP showed increased levels in patients with hyperferritinaemia compared to patients with OFI at each time point (Figure 4D). Plasminogen activator inhibitor-1 (PAI-1) can counteract the fibrinolytic system. Levels of PAI-1 were significantly elevated in patients with hyperferritinaemia compared to OFI at day 4–5 (Figure 4E). Activation of the coagulation and fibrinolytic systems eventually result in the production of D-dimers (Figure 4F). Levels of D-dimers were significantly increased in patients with hyperferritinaemia compared to patients with no hyperferritinaemia and OFI at day 2–3 and levels were significantly elevated in patients with hyperferritinaemia compared to OFI at day 4–5 and in patients without hyperferritinaemia compared to OFI at day 6–8.

The coagulation and fibrinolytic systems are highly activated in dengue patients and dengue patients with hyperferritinaemia in particular. The strongest activation was shown at day 2–3 and 4–5 after onset of fever with increased levels of vWF, TAT, PAP and D-dimer.

### Ferritin levels in a Brazilian cohort are associated with disease severity and immune activation

To confirm our findings concerning ferritin levels in the cohort from Aruba, we studied ferritin in a previously published dengue cohort obtained during the 2010 DENV outbreak in Brazil. This cohort consisted of 50 WS−, 49 WS+ and 33 severe dengue patients (More clinical details about this cohort are described in the previous publication and in Tables S1,S3 and S4 [12]).

**Figure 5:**
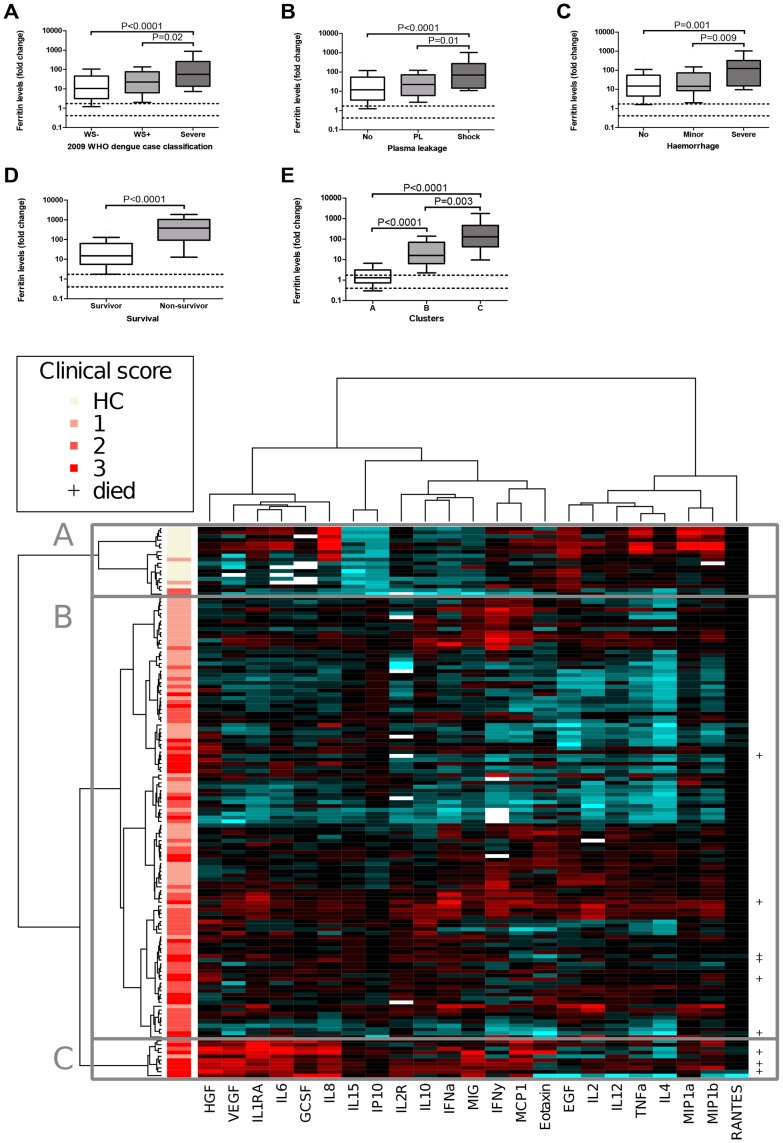
Ferritin fold change is significantly associated with clinical disease severity and a pro-inflammatory cytokine profile in the cohort from Brazil. The ferritin fold change was significantly elevated in patients with clinically severe disease as shown by classifying patients according to the 2009 WHO classification (A), occurrence of plasma leakage and shock (B), occurrence of haemorrhage (C) and survival (D). Moreover, levels were also significantly increased in Cluster C, which contains severely ill dengue patients with a pro-inflammatory cytokine profile (E). Abbreviations: WS− = non-severe dengue without warning signs. WS+ = non-severe dengue with warning signs. PL = plasma leakage. Area between two dotted horizontal lines: interquartile range from the healthy control group (N = 14). Boxplots indicate the interquartile range, the horizontal line inside the box indicates the median. The whiskers reach from the 10^th^ till the 90^th^ percentile. P-value≤0.02 is considered significant. Heatmap: This heatmap has been published previously [12]. A cluster analysis was performed with 23 cytokines, which resulted in a dendrogram indicated on the left of the heatmap. Every horizontal line indicates one patients. The vertical bar on the left of the heatmap indicates the disease severity of the patient. **A:** Cluster with mainly healthy controls and four dengue patients. **B:** Cluster with mild to moderately ill dengue virus infected patients. **C:** Cluster with severely ill dengue virus infected patients. Abbreviations: 1: non-severe dengue without warning signs. 2: non-severe dengue with warning signs. 3: severe dengue. +: patient died within 14 days after the onset of fever.

In this cohort the ferritin fold change was calculated with the same formula as described for the cohort of Aruba, because the autologous control group in Aruba was much larger (N = 45) than the healthy control group in Brazil (N = 14). The ferritin fold change was significantly elevated in patients with severe dengue according to the 2009 WHO classification, as well as in patients with shock and severe haemorrhage compared to patients with uncomplicated dengue (Figure 5A, B and C). In non-survivors levels were significantly elevated compared to survivors (Figure 5D). The absolute values of the ferritin fold change were on average higher in the Brazilian than in the Aruba cohort. This could be due to the presence of more severe disease in the cohort from Brazil and the use of a different assay.

Patients were clustered based on the expression of the determined cytokines as has been previously described (Figure 5E and heatmap) [12]. Cluster A contained mainly healthy controls, cluster B mild to moderately ill dengue patients and cluster C contained severely ill dengue patients. Severe dengue (P = 2.2×10^−16^), shock (3.4×10^−5^), severe haemorrhage (P = 0.007) and death (P = 0.03) occurred significantly more often in cluster C than the other two clusters. Cluster C showed a pro-inflammatory cytokine profile with increased expression of IL-6, IL-8, IL-10, IL-15, IL-1RA, sIL-2R, HGF, VEGF, G-CSF, MCP-1, IP-10, and MIG. Levels of ferritin were significantly increased in cluster C compared to the other two clusters and levels were also significantly elevated in the ‘dengue’ clusters B and C compared to healthy control cluster A (Figure 5E). In summary, we can conclude that levels of ferritin were significantly associated with clinical disease severity and a pro-inflammatory cytokine profile.

## Discussion

In the cohort from Aruba, increased concentrations of ferritin were significantly associated with a confirmed dengue diagnosis and viraemia. Moreover, hyperferritinaemia in dengue was strongly associated with thrombocytopenia and increased levels of liver enzymes and both activation of the coagulation and the fibrinolytic systems. The findings were confirmed in a cohort from Brazil, in which increased levels of ferritin were associated with severe disease and a pro-inflammatory cytokine profile.

Ferritin is an acute-phase reactant and a significant amount is produced by monocytes, macrophages and hepatic cells. It has been shown that synthesis of ferritin can be induced by cytokines and iron [19], [20]. We showed that increased levels of ferritin were associated with a pro-inflammatory cytokine profile. Lipopolysaccharide (LPS) was shown to induce iron retention in human monocytic cells, which may subsequently induce ferritin expression [21]. Interestingly, increased levels of LPS have been reported in patients with dengue and were also associated with a pro-inflammatory cytokine profile, suggesting that cytokines, LPS and ferritin all play a role in immune activation in severe dengue [12], [22]. Interestingly, it has been shown in vitro that ferritin can bind to high-molecular-weight kininogen and block the release of bradykinin [23]. Bradykinin is a potent vasoactive agent and plays an important role in the induction of vascular permeability and even hypotension (reviewed in [24], [25]). This may suggest that ferritin during DENV infection is induced in an effort to protect the host.

It is well known that infectious diseases in general cause hyperferritinaemia (reviewed in [6]). We showed that even in mild dengue, the occurrence of hyperferritinaemia could serve as a discriminatory marker between dengue and other febrile illnesses. Increased levels of cytokines and LPS have been reported in several infectious diseases and therefore these mechanisms cannot solely explain these extremely high ferritin levels. Macrophages, monocytes and lymphocytes in the peripheral blood are the major target cells of DENV replication in vivo [26], [27]. Monocytes and macrophages are also important producers of ferritin and therefore direct infection and subsequent viral replication in these cells may activate them and increase the ferritin production. In agreement with this, ferritin levels in the convalescent phase correlated strongly with the viral load in the early phase. Interestingly, a high viral load in the early phase of DENV infection has previously been associated with the development of severe symptoms around the time of defervescence [28].

Hepatocytes can also synthesize ferritin and in our study liver enzymes were significantly elevated in patients with hyperferritinaemia. DENV replicates very well in hepatic cell lines in vitro, but whether DENV replicates well in the liver in vivo is still a matter of debate [26]. However, it is likely that liver cells are also indirectly activated by cytokines and/or activated immune cells to produce high amounts of ferritin. It has been shown that DENV infection in mice resulted in NK and CD8+ T cell infiltration of the liver [29].

HLH is characterized by extensive activation and proliferation of NK and CD8+ T cells. CD8+ T-cells can be infected by DENV in vitro [30]. Moreover, apoptosis of CD8+ T cells plays an important role in immune modulation during DENV infection [31]–[33]. sIL-2R is a marker of T-cell activation and increased levels of sIL-2R have been detected in dengue patients with severe disease [28], [34], [35]. In a previous study with patients from the Brazilian cohort, increased levels of sIL-2R were associated with mortality [12]. In this study, using the same cohort, levels of ferritin were also significantly associated with mortality, suggesting that extensive activation of monocytes and macrophages with subsequent T-cell activation may be detrimental for the host during DENV infection.

Certain HLH-criteria, such as hypertriglyceridaemia, hypofibrinogenaemia and cytopenia in at least two cell lineages were not found in this study, most probably because the patients in this cohort only suffered from uncomplicated dengue. Increased triglyceride levels and hypofibrinogenaemia have been reported in patients with dengue shock syndrome and non-survivors [36], [37]. Therefore, we cannot exclude that HLH-like disease occurs in dengue patients with severe symptoms.

In our study thrombocytopenia was strongly associated with hyperferritinaemia. Thrombocytopenia is a hallmark of DENV infection and it is hypothesized that it can be caused by binding of platelets to activated endothelial cells [38]. Platelets are most probably bound by vWF multimers, which were increased in patients with hyperferritinaemia in this study. Because the cytopenia was limited to the platelet count in DENV infection, it is not very likely that phagocytosis by highly activated macrophages is the cause of thrombocytopenia as in the case of HLH.

Coagulopathy is one of the criteria of HLH and also described in severe dengue (reviewed in [39]). In our cohort, the coagulation and fibrinolytic systems were highly activated in patients with hyperferritinaemia at day 2–3 and 4–5 after the onset of fever resulting in increased levels of vWF, TAT, PAP and D-dimer. Levels of TAT were increased in patients with hyperferritinaemia, while levels of peak thrombin were decreased. TAT is a marker of thrombin generation in vivo, while peak thrombin is a marker for the potential of plasma to generate thrombin in vitro. From these results we may conclude that coagulation activation, thrombin formation and the consumption of coagulation factors decrease the ex vivo capacity for clotting during DENV infection, which may result in clinical bleeding symptoms. In addition to activation of the coagulation cascade, increased levels of PAP, PAI-1 and D-dimer showed that the fibrinolytic system was also highly activated in patients with hyperferritinaemia.

Based on the collective results presented in the manuscript, hyperferritinaemia can be considered as a clinical marker for DENV infection, which can discriminate between dengue and other febrile illness. Moreover, ferritin can also serve as a marker for highly active disease resulting in extensive immune activation, coagulation disturbances and severe clinical symptoms. Therefore, we suggest that patients with hyperferritinaemia are monitored carefully, as they are at higher odds to develop severe disease.

## Supporting Information

Checklist S1 **STROBE checklist (Cohort Aruba).**
(DOC)

Figure S1 **The association between ferritin and viral load in the cohort from Aruba.** Absolute levels of ferritin at day 6–8 were significantly associated with the virus titer at day 2–3 (A) and 4–5 (B). The ferritin fold change at day 6–8 was also significantly associated with the virus titer at day 2–3 (C) and 4–5 (D).
(EPS)

Table S1 **Sample size cohort Brazil and Aruba.** Abbreviations: WS−: non-severe dengue without warning signs. WS+: non-severe dengue with warning signs. OFI: other febrile illnesses.
(DOCX)

Table S2 **Laboratory values cohort Aruba.** In one patient, ferritin levels were not determined. Abbreviations: WS− = non-severe dengue without warning signs. WS+ = non-severe dengue with warning signs, OFI = other febrile illness, MWU = Mann-Whitney U test, DENV = dengue virus. * = Values are in median (interquartile range).
(DOCX)

Table S3 **Baseline characteristics of the clinical classifications of the cohort from Brazil (This table has been published previously** [12] **).** Baseline characteristics of the cohort when the patients are divided according to the 2009 WHO dengue case classification, the occurrence of plasma leakage and shock and the occurrence of hemorrhagic manifestations. Abbreviations: WS−: non-severe dengue without warning signs, WS+: non-severe dengue with warning signs. * values are given in median (interquartile range).
(DOCX)

Table S4 **Clinical characteristics of the cluster analysis of the cohort from Brazil (this table has been published previously** [12] **).** Clinical manifestations of patients divided in the three clusters. Abbreviations: HC: healthy control, WS−: non-severe dengue without warning signs, WS+: non-severe dengue with warning signs, PL: plasma leakage. * values are given in median (interquartile range).
(DOCX)
