# Supplementary material for: Hyperferritinaemia in Dengue Virus Infected Patients Is Associated with Immune Activation and Coagulation Disturbances
Source: PLoS Negl Trop Dis. 2014 Oct 9;8(10):e3214. doi: 10.1371/journal.pntd.0003214 (PMC4191960; doi:10.1371/journal.pntd.0003214)
Supplement: Table S1 — Sample size cohort Brazil and Aruba. Abbreviations: WS−: non-severe dengue without warning signs. WS+: non-severe dengue with warning signs. OFI: other febrile illnesses. (DOCX) [file pntd.0003214.s003.docx]

|  |  | Aruba | | | Brazil |
| --- | --- | --- | --- | --- | --- |
| **Figure 1** |  | Day  2-3 | Day  4-5 | Day  6-8 | Cross-sectional |
| 2009 WHO | WS- | 11 | 15 | 14 |  |
|  | WS+ | 16 | 21 | 22 |  |
| Hospitalization | Outpatients | 19 | 24 | 23 |  |
|  | Hospitalized | 8 | 12 | 13 |  |
|  | OFI | 12 | 16 | 13 |  |
| **Figure 2** |  |  |  |  |  |
| Viraemia | Not viraemic | 7 | 7 | 7 |  |
|  | Viraemic | 20 | 26 | 25 |  |
| **Figure 3** |  |  |  |  |  |
| Hyperferritinaemia | No hyperferritinaemia | 20 | 21 | 23 |  |
|  | Hyperferritinaemia | 7 | 18 | 16 |  |
|  | OFI | 12 | 16 | 13 |  |
| **Figure 4** |  |  |  |  |  |
| Hyperferritinaemia | No hyperferritinaemia | 14 | 13 | 14 |  |
|  | Hyperferritinaemia | 7 | 16 | 14 |  |
|  | OFI | 11 | 15 | 12 |  |
| **Figure 5** |  |  |  |  |  |
| 2009 WHO case classification | WS- |  |  |  | 49 |
|  | WS+ |  |  |  | 49 |
|  | Severe |  |  |  | 31 |
| Plasma leakage and shock | No plasma leakage |  |  |  | 71 |
|  | Plasma leakage |  |  |  | 33 |
|  | Shock |  |  |  | 25 |
| Haemorrhage | No haemorrhage |  |  |  | 86 |
|  | Minor haemorrhage |  |  |  | 29 |
|  | Severe haemorrhage |  |  |  | 14 |
| Survival | Non-survivors |  |  |  | 9 |
|  | Survivors |  |  |  | 120 |
| Clusters | A |  |  |  | 18 |
|  | B |  |  |  | 115 |
|  | C |  |  |  | 10 |
