# Supplementary material for: Hyperferritinaemia in Dengue Virus Infected Patients Is Associated with Immune Activation and Coagulation Disturbances
Source: PLoS Negl Trop Dis. 2014 Oct 9;8(10):e3214. doi: 10.1371/journal.pntd.0003214 (PMC4191960; doi:10.1371/journal.pntd.0003214)
Supplement: Table S2 — Laboratory values cohort Aruba. In one patient, ferritin levels were not determined. Abbreviations: WS− = non-severe dengue without warning signs. WS+ = non-severe dengue with warning signs, OFI = other febrile illness, MWU = Mann-Whitney U test, DENV = dengue virus. * = Values are in median (interquartile range). (DOCX) [file pntd.0003214.s004.docx]

|  | WS- | WS+ | Statistics | No hyper- ferritinaemia | Hyper-ferritinaemia | Statistics | OFI |
| --- | --- | --- | --- | --- | --- | --- | --- |
| **Day 2-3** | | | | | | | |
|  | N=12 | N=17 | MWU | N=20 | N=7 | MWU | N=12 |
| Viral load (copies/ml)* | 29209 (349-2089604) (N=6) | 1941 (549-17872) (N=14) | P=0.4 | 3.013 (689-3110753) (N=11) | 2009 (959-32186) (N=7) | P=0.9 | NA |
| Platelet count (x 10^9^ cells /mm^3^)* | 196 (153-275) | 155 (105-169) | P=0.03 | 172 (157-266) | 86 (74-165) | P=0.003 | 239 (164-269) |
| Leukocyte count (cells/mm^3^)* | 4.4 (2.8-8.0) | 4.1 (2.8-6.4) | P=0.7 | 4.6 (2.9-7.1) | 4.1 (2.4-5.1) | P=0.7 | 5.8 (4.5-7.2) |
| Haematocrit (%)* | 44 (41-44) | 41 (40-45) | P=0.6 | 42 (40-44) | 44 (40-45) | P=0.5 | 39 (35-45) |
| ASAT (U/L)* | 35 (22-65) | 43 (27-53) | P=0.7 | 31 (22-47) | 56 (43-227) | P=0.003 | 24 (22-35) |
| ALAT (U/L)* | 37 (18-91) | 28 (20-38) | P=0.6 | 24 (18-41) | 36 (28-154) | P=0.09 | 21 (16-29) |
| CRP (mg/L)* | 13 (5-54) | 12 (10-29) | P=0.9 | 12 (7-32) | 16 (11-31) | P=0.5 | 12 (7-44) |
| **Day 4-5** | | | | | | | |
|  | N=15 | N=24 | MWU | N=21 | N=18 | MWU | N=16 |
| Viral load (copies/ml)* | 876 (113-4269) (N=9) | 2365 (395- 13411) (N=14) | P=1.0 | 876 (193-20476) (N=9) | 2293 (211-5850) (N=14) | P=0.3 | NA |
| Platelet count (x 10^9^ cells /mm^3^)* | 170 (126-237) | 113 (68-180) | P=0.01 | 186 (139-230) | 88 (47-1320 | P<0.0001 | 209 (145-272) |
| Leukocyte count (cells/mm^3^)* | 4.7 (3.8-7.4) | 3.7 (2.5-5.2) | P=0.1 | 4.8 (2.9-7.6) | 3.8 (2.6-5) | P=0.2 | 5 (4.1-7.3) |
| Haematocrit (%)* | 43 (39-46) | 42 (38-47) | P=0.9 | 40 (38-44) | 46 (42-47) | P=0.02 | 39 (35-44) |
| ASAT (U/L)* | 37 (22-85) | 70 (33-115) | P=0.07 | 30 (23-40) | 95 (81-213) | P<0.0001 | 25 (19-33) |
| ALAT (U/L)* | 33 (22-77) | 44 (26-93) | P=0.8 | 26 (21-45) | 67 (42-153) | P<0.0001 | 21 (17-36) |
| CRP (mg/L)* | 12 (5-30) | 8 (5-18) | P=0.7 | 10 (5-37) | 7 (5-20) | P=0.7 | 9 (5-21) |
|  |  |  |  |  |  |  |  |

| **Day 6-8** | | | | | | | |
| --- | --- | --- | --- | --- | --- | --- | --- |
|  | N=15 | N=25 | MWU | N=23 | N=16 | MWU | N=13 |
| Viral load (copies/ml)* | 109 (59-207) (N=5) | 526 (89- 3970) (N=9) | P=0.1 | 110 (58-468) (N=8) | 471 (93-2881) (N=5) | P=0.2 | NA |
| Platelet count (x 10^9^ cells /mm^3^)* | 218 (141-283) | 132 (64-195) | P=0.005 | 200 (141-283) | 115 (53-171) | P=0.001 | 225 (167-290) |
| Leukocyte count (cells/mm^3^)* | 5.7 (3.9-7.5) | 4.8 (3.6-6.5) | P=0.4 | 5 (4.1-7.5) | 5 (3.5-6.7) | P=0.5 | 6.3 (4.9-8.2) |
| Haematocrit (%)* | 41 (39-46) | 41 (39-44) | P=0.7 | 39 (37-42) | 45 (41-48) | P=0.001 | 39 (34-43) |
| ASAT (U/L)* | 42 (22-98) | 109 (34-147) | P=0.07 | 32 (22-55) | 127 (107-223) | P<0.0001 | 24 (19-32) |
| ALAT (U/L)* | 38 (22-95) | 76 (25-145) | P=0.4 | 28 (19-74) | 142 (79-202) | P<0.0001 | 19 (15-34) |
| CRP (mg/L)* | 10 (5-33) | 5 (5-7) | P=0.04 | 6 (5-12) | 5 (5-16) | P=0.6 | 5 (5-9) |
|  |  |  |  |  |  |  |  |
