# Supplementary material for: Hyperferritinaemia in Dengue Virus Infected Patients Is Associated with Immune Activation and Coagulation Disturbances
Source: PLoS Negl Trop Dis. 2014 Oct 9;8(10):e3214. doi: 10.1371/journal.pntd.0003214 (PMC4191960; doi:10.1371/journal.pntd.0003214)
Supplement: Table S3 — Baseline characteristics of the clinical classifications of the cohort from Brazil (This table has been published previously [12] ). Baseline characteristics of the cohort when the patients are divided according to the 2009 WHO dengue case classification, the occurrence of plasma leakage and shock and the occurrence of hemorrhagic manifestations. Abbreviations: WS−: non-severe dengue without warning signs, WS+: non-severe dengue with warning signs. * values are given in median (interquartile range). (DOCX) [file pntd.0003214.s005.docx]

| **2009 WHO dengue case classification** | | | |
| --- | --- | --- | --- |
|  | **WS- (N=50)** | **WS+ (N=49)** | **Severe (N=33)** |
| Sex | 52% male | 61,2% male | 39,4% male |
| Age* | 44 (28-57,5) | 13,5 (9,25-30,25) | 35,5 (15-58,5) |
| Day of fever* | 3 (3-5) | 5 (4-7) | 6 (4-7) |
|  |  |  |  |
| **Plasma leakage and shock** | | | |
|  | **No (N=74)** | **Plasma leakage (N=33)** | **Shock (N=25)** |
| Sex | 52,7% male | 66,7% male | 32,0% male |
| Age* | 38 (23-55,25) | 13 (8-26) | 42 (12-62,5) |
| Day of fever* | 4 (3-6) | 5 (3-8) | 5,5 (4-7) |
|  |  |  |  |
| **Hemorrhage** | | | |
|  | **No (N=87)** | **Minor bleeding (N=29)** | **Severe bleeding (N=16)** |
| Sex | 54,0% male | 51,7% male | 43,8% male |
| Age* | 38 (16-55) | 22 (12-59) | 31 (9-45) |
| Day of fever* | 4 (3-6) | 6 (4-9) | 4 (3,5-7) |
