# Supplementary material for: Hyperferritinaemia in Dengue Virus Infected Patients Is Associated with Immune Activation and Coagulation Disturbances
Source: PLoS Negl Trop Dis. 2014 Oct 9;8(10):e3214. doi: 10.1371/journal.pntd.0003214 (PMC4191960; doi:10.1371/journal.pntd.0003214)
Supplement: Table S4 — Clinical characteristics of the cluster analysis of the cohort from Brazil (this table has been published previously [12] ). Clinical manifestations of patients divided in the three clusters. Abbreviations: HC: healthy control, WS−: non-severe dengue without warning signs, WS+: non-severe dengue with warning signs, PL: plasma leakage. * values are given in median (interquartile range). (DOCX) [file pntd.0003214.s006.docx]

| **Cluster** | **A** | | | | **B** | | | | **C** | | | | **Fisher's exact test** |
| --- | --- | --- | --- | --- | --- | --- | --- | --- | --- | --- | --- | --- | --- |
|  | **N=18** | | | | **N=115** | | | | **N=10** | | | |  |
| **Age (years)*** | **26,5 (22-35)** | |  |  | **31,5 (13-49)** | |  |  | **45 (14-63)** | |  |  |  |
| **Sex** | **38,9% male** | |  |  | **53,0 % male** | |  |  | **30,0% male** | |  |  |  |
| **Day of fever*** | **6 (4-11)** |  |  |  | **4 (3-6)** |  |  |  | **4 (3-5)** |  |  |  |  |
| **2009 WHO dengue case classification** | **77,8,0% (N=14) HC** | **11,1% (N=2) WS-** | **11,1% (N=2) WS+** |  |  | **40,0% (N=46) WS-** | **39,1% (N=45) WS+** | **20,9% (N=24) Severe** |  | **10,0% (N=1) WS-** | **20,0% (N=2) WS+** | **70,0% (N=7) Severe** | **Severe dengue P= 2.2 X 10^-16^** |
| **Survival** | **77,8,0% (N=14) HC** | **22,2% (N=4) Survived** |  |  |  | **94,8% (N=109) Survived** | **5,2% (N=6) Died** |  |  | **70,0% (N=7) Survived** | **30,0% (N=3) Died** |  | **Death**  **P=0.03** |
| **Hemorrhage** | **77,8,0% (N=14) HC** | **16,7% (N=3) NO** | **5,6% (N=1) Minor** |  |  | **68,7% (N=79) NO** | **22,6% (N=26) Minor** | **8,7% (N=10) Severe** |  | **40,0% (N=4) NO** | **20,0% (N=2) Minor** | **40,0% (N=4) Severe** | **Severe hemorrhage P=0.007** |
| **Plasma leakage and shock** | **77,8,0% (N=14) HC** | **16,7% (N=3) NO** | **5,6% (N=1) PL** |  |  | **57,4% (N=66) NO** | **27,0% (N=31) PL** | **15,7 % (N=18) Shock** |  | **10,0% (N=1) NO** | **20,0% (N=2) PL** | **70,0% (N=7) Shock** | **Shock P= 3.4 X 10^-5^** |
|  |  |  |  |  |  |  |  |  |  |  |  |  |  |
